# Evolution of lymph node staging in esophageal squamous cell carcinoma: insights from lymph node metastatic stations

**DOI:** 10.3389/fonc.2025.1621862

**Published:** 2025-07-30

**Authors:** Kexun Li, Simiao Lu, Jie Mao, Yongtao Han, Lin Peng, Xuefeng Leng

**Affiliations:** ^1^ Department of Thoracic Surgery, Sichuan Clinical Research Center for Cancer, Sichuan Cancer Hospital & Institute, Sichuan Cancer Center, Affiliated Cancer Hospital of University of Electronic Science and Technology of China (Sichuan Cancer Hospital), Chengdu, China; ^2^ Department of Thoracic Surgery I, Key Laboratory of Lung Cancer of Yunnan Province, Yunnan Cancer Hospital, The Third Affiliated Hospital of Kunming Medical University (Yunnan Cancer Hospital), Kunming, China; ^3^ School of Public Health, Chongqing Medical University, Chongqing, China

**Keywords:** esophageal squamous cell carcinoma, pathological n-category, lymph node, prognosis, UICC/AJCC, JES

## Abstract

Esophageal squamous cell carcinoma (ESCC) is a globally prevalent malignancy with distinct regional variations, particularly concentrated in East Asia, including China and Japan. The Union for International Cancer Control/American Joint Committee on Cancer (UICC/AJCC) system offers simplicity but lacks emphasis on anatomical specificity, such as upper mediastinal metastases. Conversely, the Japan Esophageal Society (JES) system provides detailed regional stratification, aiding preoperative planning and tailored therapy, though it demands greater clinical expertise. This review evaluates the current state of pathological lymph node staging systems for ESCC, comparing the UICC/AJCC and JES approaches and exploring emerging trends in China toward station-based staging.

## 1 Introduction

Esophageal squamous cell carcinoma (ESCC) remains one of the most common and lethal malignancies worldwide, with marked geographical variation in incidence and outcomes. In particular, ESCC accounts for the vast majority of esophageal cancers in East Asia, with especially high incidence reported in China and Japan (1–3). Global survival data from the third iteration of the CONCORD study (CONCORD-3), which included 37.5 million cancer cases from 71 countries diagnosed between 2000 and 2014, underscore these regional disparities. Between 2010 and 2014, Japan achieved the highest 5-year survival rate for ESCC at 36%, whereas most European and North American nations reported rates below 20%, and the United States reached only 20% (4, 5). More recent Japanese data from the National Cancer Center (2021) indicate that 5-year survival for ESCC has further improved to 47.5%, and a nation-wide surgical series by Watanabe M et al. (2021) demonstrated a 59.3% 5-year survival after curative esophagectomy (6). In China, the latest figures released by the National Cancer Center report a 5-year survival rate of 30.3% for ESCC patients, reflecting substantial progress from earlier decades (3). These improvements are largely attributable to the refinement of multidisciplinary treatment approaches, combining surgery with radiotherapy, chemotherapy, and immunotherapy, as well as to advances in surgical technique and perioperative care (7–10).

Lymph node (LN) metastasis is a critical determinant of prognosis in ESCC and is influenced by factors such as tumor location, histological differentiation, depth of invasion, and the use of neoadjuvant therapy. Yet the precise patterns of nodal spread remain incompletely characterized, and optimal nodal staging methods continue to evolve (11–15). Currently, two principal pathological N-classification systems are in widespread use. The UICC/AJCC TNM staging system categorizes nodal disease solely by the number of positive LNs, offering simplicity and ease of application, this approach also adopted in the Chinese Society of Clinical Oncology (CSCO) ESCC guidelines. However, this numeric method does not distinguish between nodal stations, particularly neglecting high-risk regions such as the upper mediastinum. In contrast, the 11th edition of the Japan Esophageal Society (JES) staging system classifies nodal involvement according to precise anatomical zones, reflecting the Japanese view that LN metastasis in ESCC is predominantly a regional process amenable to tailored surgical and adjuvant strategies (7–10). While the JES system provides superior granularity for preoperative assessment and individualized treatment planning, it demands a higher level of anatomical and pathological expertise, which may limit its generalizability in routine practice.

It should be emphasized that in the reference data of the 8th edition of UICC/AJCC tumor TNM staging systems, the data from European and American countries account for 70%, while the data from Asian countries only account for 20% (16). This discrepancy raises important considerations, as esophageal cancer patients in Western countries predominantly present with adenocarcinoma, whereas squamous cell carcinoma is more prevalent in China and Japan. Consequently, there is ongoing debate about whether the UICC/AJCC tumor TNM staging systems, which were primarily developed with adenocarcinoma as the focus, can adequately address the unique characteristics and clinical needs of squamous cell carcinoma in East Asia. Tailoring staging criteria to better reflect the regional epidemiology and biological behavior of ESCC could potentially enhance treatment planning and improve patient outcomes in these regions. Therefore, continuous evaluation and potential adaptation of staging systems are essential for ensuring that they serve the diverse global patient populations effectively.

Against this backdrop, there is growing interest,particularly in China,in developing station-based or hybrid nodal staging schemes that combine numerical and anatomical information (17–21). Such innovations aim to capture the complex biology of ESCC nodal spread and to enhance prognostic accuracy, aligning with incremental updates to the AJCC/UICC framework (22). In this review, we examine the evolution of pathological lymph node staging in ESCC, compare the UICC/AJCC and 11^th^ JES methodologies, and highlight emerging trends toward station-based classification, with the goal of informing future international consensus and optimizing patient outcomes.

## 2 Sources and selection criteria

We conducted a literature search in PubMed and Embase in January and October 2024 using keywords including “esophageal squamous cell carcinoma”, “lymph node staging”, “UICC/AJCC staging” and “JES staging”. The search was limited to studies published in English between January 2010 and March 2025. Relevant publications prior to 2010 were included if they provided foundational insights into lymph node staging or ESCC. We excluded non-peer-reviewed articles, case reports, and case series. Additional high-quality references identified from the bibliographies of retrieved articles were also reviewed and included if relevant.

## 3 Challenges facing current mainstream staging systems

The pathways of lymph node metastasis in ESCC are complex and multifactorial, involving tumor cell dissemination mechanisms and host physiological responses. Generally, ESCC spreads via direct invasion, lymphatic channels, and hematogenous routes. Direct invasion refers to tumor cells infiltrating the esophageal wall and adjacent tissues, impacting nearby lymph nodes—this mode is common in locally advanced, aggressive tumors with significant invasion. Lymphatic spread is considered the primary pathway, with tumor cells entering local lymphatic vessels and subsequently metastasizing to regional and distant lymph nodes (23–25). This process is influenced by factors such as tumor biology, lymphatic vessel integrity, and the host’s immune response.

Research indicates that metastatic involvement of specific lymph node stations significantly affects prognosis. For example, metastases in certain zones, such as the para-recurrent laryngeal nerve nodes (e.g., 106recR), the upper mediastinal, and the lower thoracic nodes, are associated with poorer survival outcomes (11–13, 26, 27). Notably, the efficacy index (EI) of lymph node dissection varies according to nodal location; studies from Japan and China highlight that dissection of high-yield stations like the upper mediastinal nodes can improve staging accuracy and survival (11, 12).

The traditional numerical N-staging system, such as that proposed by the AJCC and UICC, categorizes nodal involvement solely based on the number of positive lymph nodes, offering simplicity but neglecting anatomical nuances. While this approach facilitates widespread application, it does not capture the biological and prognostic significance of specific nodal stations, especially given the regional variability in lymphatic drainage pathways. In contrast, anatomical-based systems, like the 11^th^ JES classification, recognize the importance of nodal zones, providing more precise staging that can guide tailored surgical strategies and postoperative management (8, 28, 29).

Despite its advantages, station-based staging presents several challenges. It requires detailed knowledge of lymphatic anatomy, high-quality imaging, and meticulous pathological assessment, which are not universally available, especially in centers with limited resources. Moreover, the heterogeneity in surgical practices and lymphadenectomy extent complicates standardization. The recent update in the 12^th^ JES classification, aligning with the AJCC system by adopting the number-based staging, reflects efforts to reconcile detailed anatomical insights with practical applicability. Nevertheless, integrating regional lymphatic patterns into a universally accepted staging system remains an ongoing challenge, especially considering variations in tumor location, patient anatomy, and surgical methods (e.g., intraoperative thoracic duct ligation and different locations of anastomosis). So, refining current staging systems to address these multifaceted challenges will be crucial for optimizing outcomes in ESCC patients worldwide (28, 29).

A large-scale study focusing on pN1-stage esophageal squamous cell carcinoma (ESCC) patients revealed important nuances: among pN1 patients, those with a single positive lymph node had significantly better survival than those with two positive nodes (HR: 0.67, 95% CI: 0.55–0.81, P=0.0001), a difference that persisted after propensity score matching (HR: 0.76, 95% CI: 0.61–0.94, P=0.0027). Crucially, when both positive nodes were located within the same lymph node station, survival outcomes were not statistically different from those with only one positive node (HR: 0.88, 95% CI: 0.65–1.18, P=0.5736; after matching, HR: 0.52, 95% CI: 0.34–0.78, P=0.2258). In contrast, patients with two positive nodes located in different anatomical lymph node stations experienced significantly worse survival than those with a single positive node (HR: 0.61, 95% CI: 0.49–0.75, P<0.0001; after matching, HR: 0.72, 95% CI: 0.56–0.93, P=0.0037) (17).

These findings suggest that, within the pN1 category, both the quantity and anatomical distribution of metastatic lymph nodes independently influence patient prognosis. Specifically, multi-station metastases are associated with a poorer survival outlook compared to multi-node involvement within a single station (18–20). This observation is consistent with the underlying biology of ESCC, where lymphatic dissemination typically follows well-defined anatomical pathways, and cross-regional spread may signify more aggressive tumor behavior and an increased likelihood of distant metastasis. Consequently, these results support the implementation of a refined, station-based nodal staging system. Such an approach could enhance risk stratification, inform postoperative adjuvant therapy decisions, and improve individualized patient management.

## 4 Insights from Chinese esophageal surgeons

Building on these N1-based study, current researches focusing on the number of metastatic lymph nodes may warrant re-evaluation (18). While AJCC/UICC staging offers simplicity and facilitates broader implementation, its ability to accurately inform prognosis is increasingly being questioned. Emerging evidence from Chinese cohorts demonstrates the superiority of station-based nodal (nN) staging systems over traditional numeric pN categories in prognostic stratification for ESCC (18–20).

In a recent large-scale analysis, the area under the curve (AUC) values for the nN staging system at 1, 3, and 5 years were 0.658, 0.679, and 0.691, respectively. For the UICC/AJCC pN staging system, the corresponding AUCs were 0.649, 0.673, and 0.682. In an independent validation cohort, nN staging yielded AUCs of 0.655, 0.714, and 0.708 at 1-, 3-, and 5-year intervals, consistently outperforming the pN system, which recorded AUCs of 0.662, 0.709, and 0.702 at the same time points. Time-dependent ROC analysis uniformly demonstrated that the AUCs for nN staging surpassed those of pN staging at every interval, with the greatest predictive advantage observed at five years (AUC: nN, 0.691 vs. pN, 0.682 in the training cohort). This trend was mirrored in the validation cohort, where nN staging maintained superior AUC values across all time points. Concordance index (C-index) analysis further substantiated the prognostic accuracy of station-based nN staging, which achieved a higher C-index than pN staging in both the training cohort (nN: 0.638 vs. pN: 0.633) and the validation cohort (nN: 0.658 vs. pN: 0.657). Additionally, likelihood ratio, Wald, and log-rank tests all demonstrated significantly better performance for nN staging compared to pN staging (all P < 0.001) (19).

Similarly, a retrospective study of 1,351 patients undergoing curative esophagectomy for ESCC reported that station-based lymph node staging systems conferred clear prognostic advantages over the UICC/AJCC pN classification. These findings collectively suggest that integrating anatomical lymph node station information enhances the prognostic discriminatory power and clinical applicability of nodal staging in ESCC, particularly for populations with distinct tumor biology and metastatic patterns, such as those seen in China (18). Station-based nodal classification not only aligns more closely with the patterns of tumor spread observed in East Asian populations but also provides a more nuanced risk stratification for postoperative management.

## 5 Discussion and limitations in current research

Despite remarkable progress in LN staging for ESCC and increasing advocacy for more refined staging systems in recent years, several key limitations and challenges continue to impede the widespread adoption and further optimization of these approaches.

Firstly, most of the evidence supporting station-based LN staging systems is derived from retrospective, single-center, or regionally focused studies. The generalizability of these findings may be limited by institutional biases, regional healthcare resources, and variations in data quality. Large-scale, prospective, multicenter studies are still lacking, making it difficult to draw universally applicable conclusions regarding the superiority and clinical utility of station-based over traditional number-based staging systems.

Secondly, even within East Asia, significant heterogeneity exists in surgical technique and practice. Variations in the extent of lymphadenectomy (such as two-field versus three-field dissection) (30, 31), approaches to thoracic duct management, and intraoperative identification of LN stations are common. The lack of standardized protocols for LN dissection and station assignment complicates outcome comparisons across institutions and studies (32–35). One of the major differences between esophageal cancer (EC) and other GI cancers relates to lymphatics. The esophagus has a rich and anastomosing network of lymphatics that interconnect in circumferential and longitudinal plexuses. This often comes into play in EC lymphatic spread whereby GEJ, distal, mid and proximal esophageal cancer can spread to other regions locally. This can involve perigastric, periesophageal, paraaortic, mediastinal and cervical LNs. It is generally believed that lymphatic involvement is an early event in EC. Tumor microfoci can sometimes be detected at the proximal margin, particularly within mural lymphatics, due to the extensive and anastomosing lymphatic plexuses present in the esophagus.

Thirdly, prognosis in ESCC is influenced by a multitude of factors beyond LN metastases. Lifestyle factors, as well as the timely administration of adjuvant therapy after surgery or the implementation of standardized neoadjuvant therapy protocols, can significantly impact patient outcomes. The interplay between these factors and LN status is complex and has not been fully elucidated in current staging paradigms (36–39).

Fourth, the impact of neoadjuvant therapy on LN staging accuracy remains a significant research challenge. Preoperative chemotherapy, radiotherapy, or immunotherapy can result in substantial tumor and LN regression, potentially leading to pathological downstaging and underestimation of the initial disease burden. This could affect the prognostic value of both number-based and station-based staging systems. Future studies should explore how to best integrate the pathological changes induced by neoadjuvant treatments into evolving LN staging frameworks (40).

Additionally, current staging models rarely incorporate multidimensional factors such as LN anatomical location, size, extranodal extension, or molecular and immunological characteristics in a unified manner. While station-based systems primarily focus on anatomical dissemination, emerging evidence suggests that combining quantitative parameters (number and distribution of positive LNs), qualitative features (extranodal extension), and biological markers could further enhance prognostic stratification and support personalized treatment strategies.

Finally, a global consensus on the optimal LN staging system for ESCC remains elusive. The divergence between Western (AJCC/UICC) and Eastern (JES or station-based) staging paradigms is closely related to differences in tumor histology.

## 6 Future directions and perspectives

Looking forward, the evolution of LN staging in ESCC is poised to benefit from several key advancements aimed at addressing the limitations of current systems and optimizing patient outcomes mainly in East Asia.

First, there is a clear need for large-scale, prospective, multicenter studies that comprehensively evaluate the prognostic and therapeutic value of station-based and hybrid LN staging systems. Such studies should strive for international collaboration to encompass diverse patient populations, surgical practices, and healthcare settings. The establishment of shared databases and standardized protocols for lymphadenectomy, nodal station assignment, and pathological assessment will be essential to minimize bias and enhance the reproducibility of findings.

Second, as surgical and perioperative techniques continue to advance, the development of consensus guidelines for the extent of lymphadenectomy and the definition of nodal stations is crucial. International working groups involving both Eastern and Western experts could help harmonize anatomical terminology and refine LN maps, ultimately facilitating data sharing and direct comparison across regions.

Third, integrating multi-dimensional clinical, molecular, and imaging data into LN staging holds promise for more precise, individualized risk stratification. Incorporating factors such as LN size, extranodal extension, spatial distribution, and relevant genomic or immunological biomarkers could create a more comprehensive and biologically relevant staging model. Advances in artificial intelligence and machine learning may further accelerate the development of predictive algorithms that synthesize these complex data streams and guide clinical decision-making.

Lastly, achieving a global consensus on LN staging for ESCC remains a high priority. This effort should be informed by robust, regionally inclusive evidence and should account for differences in tumor biology, healthcare resources, and clinical practice patterns. The creation of a universally accepted, hybrid staging system that combines anatomical and quantitative information—while also being adaptable to local contexts—may represent the optimal way forward. In summary, the next generation of LN staging for ESCC will be shaped by international collaboration, technological innovation, and a commitment to individualized patient care.
